# DDX3X Multifunctionally Modulates Tumor Progression and Serves as a Prognostic Indicator to Predict Cancer Outcomes

**DOI:** 10.3390/ijms21010281

**Published:** 2019-12-31

**Authors:** Tsung-Chieh Lin

**Affiliations:** Genomic Medicine Core Laboratory, Chang Gung Memorial Hospital, Linkou, Taiwan; tclin1980@cgmh.org.tw; Tel.: +886-3-328-1200 (ext. 7722)

**Keywords:** DDX3X, cancer progression, clinical outcomes

## Abstract

DEAD (Asp-Glu-Ala-Asp) box polypeptide 3, X-Linked (DDX3X), also known as DDX3, is one of the most widely studied and evolutionarily conserved members of the DEAD-box RNA helicase subfamily, and has been reported to participate in several cytosolic steps of mRNA metabolism. DDX3X facilitates the translation of specific targets via its helicase activity and regulates factors of the translation initiation complex. Emerging evidence illustrates the biological activities of DDX3X beyond its originally identified functions. The nonconventional regulatory effects include acting as a signaling adaptor molecule independent of enzymatic RNA remodeling, and DDX3X exhibits abnormal expression in cancers. DDX3X interacts with specific components to perform both oncogenic and tumor-suppressive roles in modulating tumor proliferation, migration, invasion, drug resistance, and cancer stemness in many types of cancers, indicating the need to unravel the associated molecular mechanisms. In this review article, we summarized and integrated current findings relevant to DDX3X in cancer research fields, cytokines and compounds modulating DDX3X’s functions, and the released transcriptomic information and cancer patient clinical data from public databases. We found evidence for DDX3X having multiple impacts on cancer progression, and evaluated DDX3X expression levels in a pancancer panel and its associations with patient survival in each cancer-type cohort.

## 1. Introduction

**Figure 1:**
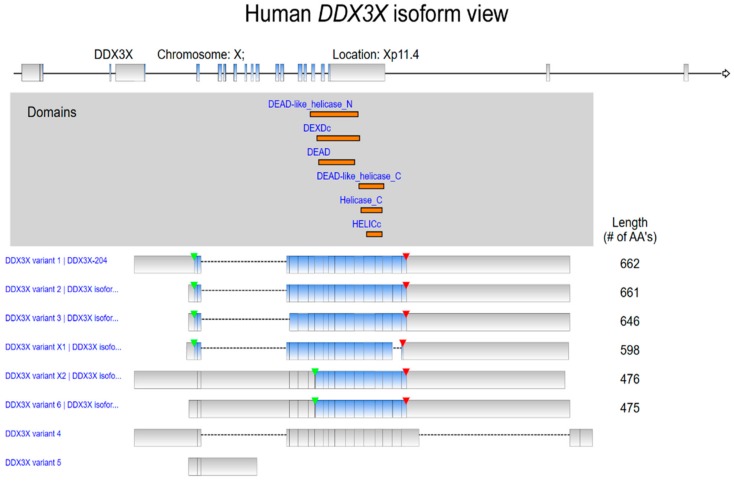
Human DDX3X isoform view from RefSeq. Data were analyzed by using ingenuity pathway analysis (IPA). Protein domains of various DDX3X isoforms are marked and located by orange color. The start of transcription and the position of a stop codon are indicated by green and red arrowheads, respectively.

DEAD (Asp-Glu-Ala-Asp) box polypeptide 3, X-Linked (DDX3X) is a member of the DEAD-box family of RNA helicases and has been reported to be involved in double-stranded RNA unwinding [1], pre-mRNA splicing [2], RNA export [3], transcription [4], and translation [5,6,7]. Recently, the helicase activity of DDX3X was reported to repress the repeat-associated non-AUG translation of GGGGCC repeats in C9ORF72 via direct binding, thereby reducing the aberrant accumulation of dipeptide repeat that might be likely to ameliorate amyotrophic lateral sclerosis (ALS) and frontotemporal dementia (FTD) [8]. Various previously identified DDX3X variants were represented, along with distinct protein domains (Figure 1) [9]. Actually, prevalence of *DDX3X* somatic mutations has been found in cancer patients [10,11,12]. In a chronic lymphocytic leukemia cohort, all identified *DDX3X* mutations were truncating mutations, including nonsense mutations or frameshift indels [13]. Twenty-one out of 105 cases of natural killer/T-cell lymphoma also showed recurrent *DDX3X* somatic mutations leading to impaired RNA-unwinding function [14]. Somatic evolution due to the accumulation of genomic changes may alter cancer development and progression, including drug resistance and cell proliferation. In a gene gravity model, tumor genomes harboring DDX3X nonsynonymous somatic mutations appear to have high mutation density [15]. Emerging evidence indicates the critical regulatory role of DDX3X in cancer progression. Due to its complicated function in RNA metabolism, DDX3X has gained increasing attention for its biological functions in various types of cancers and has been shown to modulate cancer progression in a complex manner. This complexity was further increased by evidence revealing that DEAD box proteins generally do not function alone but instead act as components of multiprotein complexes [16]. The exact function of DDX3X is affected by its interacting partners and is tumor and/or context dependent [17]. DDX3X is characterized as a multifunctional RNA helicase that regulates RNA metabolism via direct binding with RNA targets. In breast cancer, DDX3X directly interacts with KLF4 mRNA and regulates its splicing [18]. RNA G-quadruplexes (rG4s) are a secondary structure of mRNAs known to influence posttranscriptional mechanisms involving RNAs. A recent report further showed that a systematic affinity proteomics approach identified several high-confidence interactors, including DDX3X, which could assemble into the rG4 located in the 5′-untranslated region (UTR) of the NRAS oncogene transcript. Furthermore, the interaction of 5′-UTR rG4-containing transcripts was decreased upon mutation of the DDX3X glycine-arginine (GAR) domain [19]. Both tumor-promotive and tumor-suppressive effects of DDX3X have been identified and reported. These controversial results emphasize the urgent need to clarify the prognostic value of DDX3X and to unravel the molecular mechanisms specifically involved in human cancer types. Therefore, we summarized and integrated evidence demonstrating DDX3X expression levels in a broad range of cancer types together with DDX3X-mediated effects on the regulation of several critical factors in processes related to cancer progression, including tumor proliferation, metastasis, drug resistance, and cancer stemness. In this review, we focus on the biological function of DDX3X in cancer, and further illustrate its clinical significance on a pancancer scale.

## 2. DDX3X Expression in Cancers

**Figure 2:**
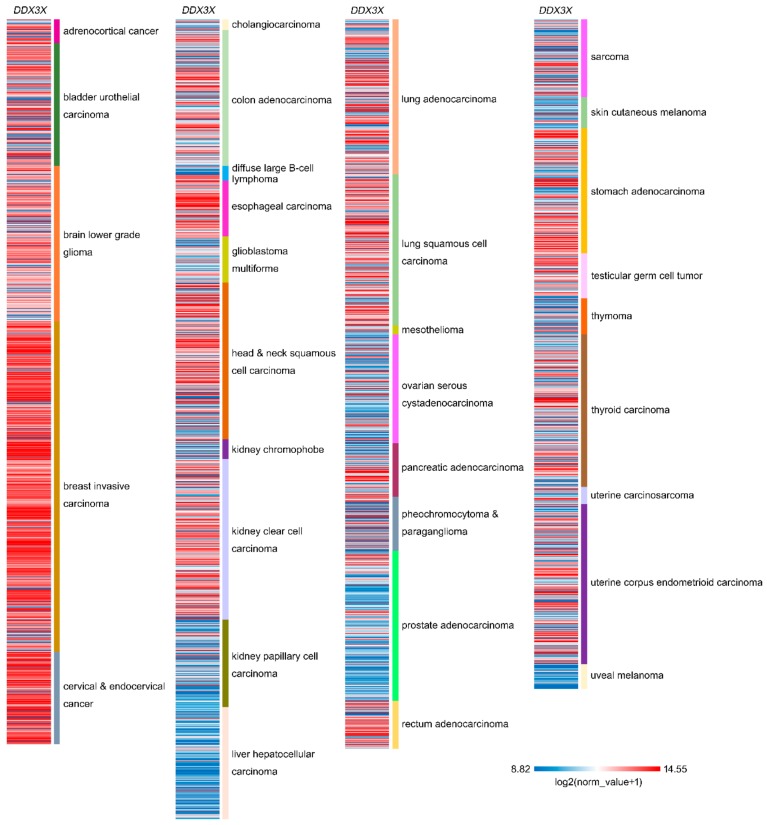
Relative DDX3X expression in a pancancer panel. In a pancancer dataset from The Cancer Genome Atlas, DDX3X expression levels were presented separately for 32 cancer types. The red color in the heat map represents genes with high expression. The blue color in the heat map represents genes with low expression.

The relative expression of DDX3X in various cancer types demonstrates its pivotal role in tumor progression. In colorectal cancer, DDX3X expression has been detected, and positive associations between DDX3 and KRAS, YAP1, and SIX2 have been observed in KRAS wild-type patients [20]. In addition, increased cytoplasmic DDX3X expression has been observed in breast cancer metastases, especially in triple-negative and high-grade cases [21]. A comparison of matched tumor and normal tissue further indicated that the DDX3 level was obviously higher in pancreatic ductal adenocarcinoma tissue than in peritumoral tissue, benign pancreatic tissue, and normal pancreatic tissue (*p* < 0.01) [22]. DDX3X overexpression has been reported in prostate cancers, and overexpression was found to be directly associated with high Gleason scores [23]. Furthermore, an investigation of DDX3X protein levels in 303 colorectal cancer samples evaluated by immunohistochemistry revealed that 39% of tumors exhibited DDX3X overexpression and that high cytoplasmic DDX3 expression appeared to associate with nuclear β-catenin expression, suggesting the potential involvement of Wnt signaling [24]. A comprehensive program analyzing the combination of multicancer transcriptomic data and matched clinical information was launched by the University of California Santa Cruz (*n* = 12,839) [25]. These transcriptomic data were mainly generated by microarray experiments and RNA sequencing (RNA-Seq) on a pancancer scale and retrieved from the public database The Cancer Genome Atlas (TCGA). The results showed DDX3X expression after normalization in various types of cancer (Figure 2). DDX3X is relatively highly expressed in stomach adenocarcinoma, kidney clear cell carcinoma, breast invasive carcinoma, cervical and endocervical cancer, esophageal carcinoma, and head and a sub-population of neck squamous cell carcinomas. Additionally, note that the lower DDX3X levels were displayed in uveal melanoma, diffuse large B-cell lymphoma, thymoma, liver hepatocellular carcinoma, kidney papillary cell carcinoma, and skin cutaneous melanoma, which indicates that DDX3X might has a dual role in cancer progression.

## 3. Correlations with Clinical Outcome

The correlation of DDX3X with clinical prognosis has been evaluated in various types of cancer. Both KRAS wild-type and mutant cohorts of colorectal cancer patients were shown to exhibit high DDX3X expression associated with poor overall survival and relapse-free survival [20]. Associations with poor overall survival and relapse-free survival have also been reported for colorectal cancer cohorts comprising 145 cases [26] and 75 cases [27]. In addition, relatively poor overall survival has been reported for a cohort of 79 breast patients with distant metastasis expressing high DDX3 (hazard ratio (HR) 1.79, *p* = 0.039) [21]. Similar observations were uncovered in another breast cancer cohort study, in which 35% of the breast cancer patients displayed high DDX3X levels that correlated with poor clinical outcomes [28]. Furthermore, the prognostic power of DDX3X has been specifically correlated with the nuclear DDX3X expression level in both breast and colorectal cancers [29]. In head and neck squamous cell carcinoma patients, the DDX3X level correlates with lymph node metastasis and a poor prognosis [30]. Another study of pancreatic ductal adenocarcinoma further showed that positive DDX3X expression was correlated with poor outcomes by Kaplan–Meier survival analysis (*p* < 0.001), and the data from Cox multivariate analysis also indicated that DDX3 was an independent poor prognostic factor in cancer patients [22]. However, several research teams have also demonstrated that DDX3X is a biomarker of a good prognosis in cancers. Patients with low DDX3 expression were reported to have worse survival and higher relapse rates than those with high DDX3X expression in a non-small cell lung cancer cohort [31]. In colorectal cancer, high DDX3X protein levels were shown to predict good overall survival and recurrence-free survival in a 221-case cohort, and the RNA expression of DDX3X had significant prognostic value when evaluated by RNA-Seq and microarray platforms [32]. Furthermore, DDX3X expression profiles determined by microarray and RNA-Seq technologies have been analyzed together with clinical follow-up data from public databases including The Human Protein Atlas/The Pathology Atlas [33,34,35,36,37], SurvExpress [38], the TCGA [25], and the Kaplan–Meier plotter database [39], which illustrates the prognostic value of DDX3X in specific cancer types (Table 1). *DDX3X* is a poor prognostic marker in cohorts of liver cancer, pancreatic cancer, breast cancer, and ovarian cancer, while patients of colorectal cancer, urothelial cancer, lung cancer, and gastric cancer expressing high *DDX3X* level associate with better outcomes. Another human genome *DDX3*-gene-coded homolog is *DDX3Y*. The correlation with cancer survival outcome is also listed for comparison (Table 2). Patients expressing high *DDX3Y* associate with poor outcomes in cohorts of thyroid cancer, lung cancer, colorectal cancer, stomach cancer, prostate cancer, and melanoma. In contrast, *DDX3Y* is a good prognostic biomarker in head and neck cancer, pancreatic cancer, and gastric cancer.

## 4. DDX3X and Cancer Cell Proliferation

The role of DD3X in normal cells was characterized by benzo[a]pyrene diol epoxide-activated DDX3X contributing to increased cell proliferation in breast epithelial cells [40]. DDX3X appears to regulate cancer proliferation and tumorigenesis, and both oncogenic and tumor suppressor roles have been uncovered. In a study, depletion of DDX3X led to the inhibition of MCF7 cell proliferation via G1 phase arrest. DDX3X was notably found to reduce the expression of the cell cycle repressor KLF4, indicating the oncogenic role of DDX3X in breast cancer [18]. In addition, DDX3 silencing in the aggressive prostate cancer cell lines DU145 and 22Rv1 results in a significant reduction in tumor clonogenicity. Targeting DDX3X through blocking its ATP binding domain with the small molecule RK-33 synergizes with radiotherapy to decrease tumor cells proliferation in vitro and in xenograft models in vivo [23]. Loss of DDX3X function induced by shRNA or RK-33 addition elicits G1 cell cycle arrest and apoptosis in lung cancer that impairs Wnt signaling [41]. In breast cancer MCF-7 and MDA-MB-231 cell models, a biological reduction in the DDX3X level by shRNA induces decreased proliferation rates and clonogenicity, while reduced tumor volume is seen in animal experiments [42]. Moreover, DDX3X silencing by siRNA causes reductions in both TCF-4 reporter activity and TCF-4-mediated modulation of downstream target genes in colorectal cancer, which leads to reduced proliferation and G1 arrest [24]. The role of the YEATS4/TCEA1/DDX3 axis in hepatocellular carcinoma progression has been explored. The DDX3X protein is stabilized by TCEA1, which promotes hepatocellular carcinoma cell proliferation and colony formation [43]. In contrast, several studies have demonstrated a critical role for DDX3X in repressing cancer cell growth. One report showed that DDX3X participated in replicative stress in the liver. In a study utilizing a hepatocyte-specific DDX3X knockout mouse model, loss of DDX3X expression elicited liver tumorigenesis and liver cell proliferation due to decreases in the expression of two DNA repair factors, DDB2 and XPA [44]. The antitumor activity of rottlerin in hepatocellular carcinoma cells has been suggested to occur in a DDX3X-dependent manner that causes cell cycle arrest in the G1 phase. Upregulation of DDX3X expression, leading to an increase in the p21 level and cyclin D1 downregulation, has been observed after rottlerin treatment [45]. Furthermore, in non-small cell lung cancer, loss of DDX3X expression by p53 inactivation results in tumor malignancy that can be seen as an increase in soft-agar growth [31]. In a similar study, a putative p53 binding site in the DDX3X promoter was identified, and the promotion of cancer cell proliferation was observed to be caused by DDX3X deregulation caused by the E6-inactivated p53 pathway [46]. Immunofluorescence data revealed DDX3X location at centrosome, and further observed its colocalization with p53 at cell cycle mitosis phase in HCT116 and U2OS cells. DDX3X silencing resulted in repression of p53 transcription and defects in chromosome alignment, leading to G2/M phase arrest and cell death [47].

## 5. DDX3X and Cancer Cell Metastasis

Emerging studies illustrating the role of DDX3X in cancer metastasis have been reported. The DDX3X expression level has been found to be positively associated with hypoxia-related proteins, including HIF1 alpha, in invasive breast cancer, suggesting a pro-metastatic role for DDX3X [48]. In a study of melanoma, DDX3X appeared to activate translational reprogramming of MITF mRNA, leading to a proliferative-to-metastatic phenotypic switch in vivo [49]. Furthermore, DDX3X specifically increases the binding of the cap-binding complex (CBC) to the uORF of ATF4 mRNA, which leads to the recruitment of eIF3 and facilitates translation in oral squamous cell carcinoma cell invasion and metastasis [30]. In particular, cytoplasmic DDX3X levels in prostate cancer tissue samples have been associated with cancer metastasis status, and repression of DDX3X via genetic and pharmacologic methods reduces cell motility [50]. Specific shRNA-mediated DDX3X reduction suppresses breast cancer metastasis in vivo [42]. In medulloblastoma, DDX3X also appears to augment cell mobility by increasing Rac1 mRNA translation and stabilizing the β-catenin protein, suggesting DDX3X has a role in Wnt signaling [51]. Furthermore, the β-catenin/ZEB1 axis is activated by DDX3X-dependent KRAS transcription to promote colorectal cancer invasion [26]. In addition, the CK1ε/Dvl2/β-catenin axis was shown to be activated upon DDX3X overexpression to increase colorectal cancer invasion and metastasis in an animal model, whereas these effects could be blocked by inhibitors of CK1ε (PF4800567) and β-catenin/TCF signaling (XAV939) [27]. Data from a recent study further showed the binding of DDX3X with circular RNA, *circ-CTNNB1*, to facilitate cancer invasion and metastasis in gastric cancer via the transactivation of *YY1* and activation of downstream β-catenin signaling [52]. However, the results from several studies have also demonstrated that DDX3X can have a role in suppressing cancer metastasis. Depletion of DDX3X expression in liver cancer cells HepG2 increased cell migration ability, and the phenotype was reversed upon restoration of tumor-suppressive miR-200b, miR-200c, miR-122, and miR-145, respectively [53]. Relevant evidence has revealed DDX3X upregulation in diosgenin-inhibited cell migration and invasion in two liver cancer cell lines, HepG2 and SMMC-7721, along with alterations in the expression of the metastasis-related biomarkers β-catenin and E-cadherin [54]. Moreover, direct modulation of DDX3X to Slug/E-cadherin signaling axis has been shown to repress invasion in lung cancer [31]. In a colorectal cancer, knocking down DDX3X expression promoted cancer cell migration and invasion, and increased tumor metastasis in an animal model via the regulation of the Snail/E-cadherin pathway [32].

## 6. DDX3X and Drug Resistance

DDX3X likely participates in imatinib-resistant chronic myeloid leukemia. Liquid chromatography-mass spectroscopy (LC-MS)/MS data indicate an increased protein level of DDX3X upon imatinib analog (SK23 and Y18) treatments designed to inhibit Bcr-Abl tyrosine kinases [55]. Lung cancer cells harboring EGFR exon 19 deletion and DDX3X cDNA have been observed to have reduced EGFR-tyrosine kinase inhibitor (TKI) sensitivity and cancer stem cell phenotypes, suggesting a role for DDX3X in drug resistance acquisition [56]. In addition, DDX3X-dependent aggressiveness and drug resistance to cetuximab treatment have been revealed to be modulated by the YAP1-SIX2 signaling axis in KRAS wild-type colorectal cancer in both cell and animal models [20]. On the other hand, the tumor suppressor role of DDX3X has been characterized: silencing of DDX3X expression in hepatocellular carcinoma HepG2 cells promoted chemoresistance to anti-cancer drugs doxorubicin and 5-fluorouracil [53].

## 7. DDX3X and Cancer Stemness

The presence of cancer stem cells is one of factors leading to cancer progression. The role of DDX3X in stem cell maintenance and embryonic carcinoma formation has been explored. DDX3X expression at an early stage of embryonic development, especially in undifferentiated stem cells, was detected. DDX3X depletion induced terminal differentiation of lineages, and the potency for teratoma formation was repressed [57]. However, reduction of DDX3X was further reported to induce self-renewal capability and co-expression of stemness gene signature in liver cancer [53]. Furthermore, the engineered DDX3X mutation in neuroepithelial stem cells isolated from Gorlin syndrome patients induced medulloblastoma in orthotopic transplantation model [58]. Those findings further support the opposite point of view regarding DDX3X-mediated cancer stemness and tumor progression.

## 8. DDX3X Modulations by Cytokines and Compounds

**Figure 3:**
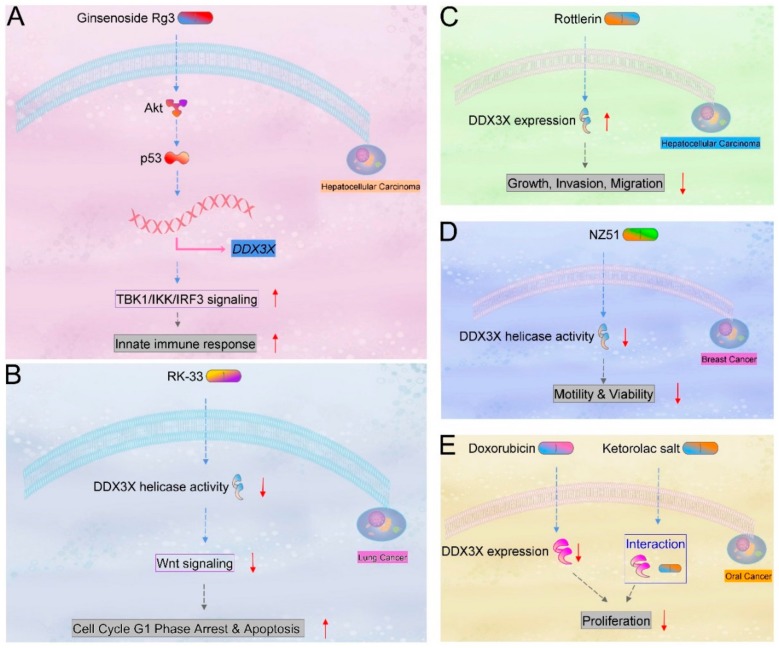
Diagram of DDX3X alternations by compounds. The changes in DDX3X activity and expression level along with downstream signaling upon (**A**) Ginsenoside Rg3, (**B**) RK-33, (**C**) Rottlerin, (**D**) NZ51, or (**E**) doxorubicin addition in various types of cancer are represented.

In addition to the DDX3X helicase activity inhibitors (Rottlerin, NZ51, RK-33) mentioned above, results from recent studies reveal that cytokines and compounds appear to regulate DDX3X and relevant phenotypes in tumor progression, which further indicates the potential values for designing cancer therapeutic strategy. FE15 and FE109 are ATP-competitive inhibitors proven to block helicase and ATPase function of DDX3X. The compounds possess activity that decreases HIV viral load in peripheral blood mononuclear cells [59]. In addition, doxorubicin is identified as a DDX3X inhibitor: the binding to unique amino acid residue Thr 198 and common amino acid residues Tyr 200 and Thr 201 has been characterized by an in-silico molecular docking approach. Treatment of doxorubicin in oral squamous cell carcinoma H357 cells showed the decrease in inorganic phosphate (Pi) release, ATP hydrolysis, DDX3X downregulation, and the anticancer activity, which was evaluated by MTT method [60]. Ketorolac salt is another bioactive compound selected from high throughput virtual screening. Ketorolac salt was found to interact with DDX3X and revealed the growth inhibitory effect in oral cancer cell model [61]. In liver hepatocellular HepG2 cells, treatment with 5-aza-2′-deoxycytidine (decitabine), SP2509, EPZ-6438 (tazemetostat), and TSA (trichostatin A) resulted in the reduced expression of DDX3X at the RNA level [62]. Furthermore, 1,3,4-thiadiazole is a novel DDX3X ATPase activity inhibitor synthesized for countering HIV-1 effects [63]. IL17-DDX3X-Zc3h12a signaling axis might modulate inflammation in cancer microenvironment. IL17 stimulus facilitated the formation of a DDX3X–CIKS complex which is required for the stabilization of Zc3h12a mRNA [64]. Zc3h12a has been pointed out to negatively regulate cellular inflammation in many ways. Zc3h12a led to the mRNA degradation of cytokine IL-6 and IL-12p40 [65,66]. In human monocyte-derived macrophages, the IL-1β, LPS- and TNF-α-dependent NF-κB, and JNK signaling pathways were blocked by Zc3h12a [67]. In liver hepatocellular cells, 5-HT treatment increased 5-HT receptor 7-dependent *DDX3X* promoter activity, along with downstream expression, to induce innate immunity against hepatitis B virus (HBV) infection [68]. Activation of innate immune response through the TBK1/IKKε/IRF3 signaling axis was also observed by the ginsenoside Rg3 stimulus, which increased Akt-p53-dependent DD3X promoter transactivation and its expression level [69]. A microarray analysis also showed fibronectin 1 could increase the expression of human DDX3X mRNA in cultured HUVEC cells [70]. AGR2, a member of the protein disulfide isomerase family, is also a proto-oncogene, and the extracellular form of AGR2 has been seen to induce tumor metastasis in various in vivo animal models. The bindings of human AGR2 protein and human DDX3X protein have been characterized, though the molecular mechanism(s) remain to be further explored [71]. Furthermore, interactions of DDX3X with TNF [72], TGFβ1 [73], and Fibronectin 1 [74], proven by affinity chromatography, have been reported. The effects of small compounds on DDX3X in various cancers are represented (Figure 3).

## 9. Discussion and Conclusions

We summarized and integrated published references and data from an in silico analysis to clarify the relative expression levels of DDX3X in multiple types of cancers. In addition to the clinical outcomes associated with DDX3X levels observed on a pancancer scale, the pivotal roles of DDX3X in modulating processes relevant to cancer progression, including cancer cell proliferation, metastasis, drug resistance, and cancer stemness, were also illustrated, along with the upstream regulators, small molecules, and cytokines, from extracellular space. Notably, the differential expression of DDX3X at the RNA level in specific cancer types further highlights the value of mechanistic studies regarding alterations in transcriptional activity and RNA stability. In contrast, evaluations of prognostic significance revealed a discrepancy in several types of cancers, indicating that the impact of DDX3X on those tumor types remains to be explored and additional evidence is required. Furthermore, the differences resulting from variations in analytic platforms and endpoint designs of studies should be considered. Actually, prognostic power is judged by the various number of cases enrolled in each cohort.

## Figures and Tables

**Table 1:** The correlation of *DDX3X* with cancer patient survival.

Symbol	Cancer Type	Prognosis	Endpoint	*p* Value	Case	Dataset	Method	Probe ID
*DDX3X*	Glioma	-	Overall survival	N.S.	153	TCGA	RNA Seq	
*DDX3X*	Thyroid Cancer	-	Overall survival	N.S.	501	TCGA	RNA Seq	
*DDX3X*	Lung Cancer	-	Overall survival	N.S.	994	TCGA	RNA Seq	
*DDX3X*	Colorectal Cancer	Good	Overall survival	0.008	597	TCGA	RNA Seq	
*DDX3X*	Head and Neck Cancer	-	Overall survival	N.S.	499	TCGA	RNA Seq	
*DDX3X*	Stomach Cancer	-	Overall survival	N.S.	354	TCGA	RNA Seq	
*DDX3X*	Liver Cancer	Poor	Overall survival	0.001	365	TCGA	RNA Seq	
*DDX3X*	Pancreatic Cancer	Poor	Overall survival	0.033	176	TCGA	RNA Seq	
*DDX3X*	Renal Cancer	-	Overall survival	N.S.	877	TCGA	RNA Seq	
*DDX3X*	Urothelial Cancer	Good	Overall survival	0.029	406	TCGA	RNA Seq	
*DDX3X*	Prostate Cancer	-	Overall survival	N.S.	494	TCGA	RNA Seq	
*DDX3X*	Testis Cancer	-	Overall survival	N.S.	134	TCGA	RNA Seq	
*DDX3X*	Breast cancer	Poor	Overall survival	0.024	1075	TCGA	RNA Seq	
*DDX3X*	Cervical Cancer	-	Overall survival	N.S.	291	TCGA	RNA Seq	
*DDX3X*	Endometrial Cancer	-	Overall survival	N.S.	541	TCGA	RNA Seq	
*DDX3X*	Ovarian Cancer	-	Overall survival	N.S.	373	TCGA	RNA Seq	
*DDX3X*	Melanoma	-	Overall survival	N.S.	102	TCGA	RNA Seq	
*DDX3X*	Breast cancer	Poor	Relapse-free survival	<0.001	3951	E-MTAB-365, E-TABM-43, GSE: 11121, 12093,	Array	201210_at
						12276, 1456, 16391, 16446, 16716, 17705, 17907,		
						18728, 19615, 20194, 20271, 2034, 20685, 20711,		
						21653, 2603, 26971, 2990, 31448, 31519, 32646,		
						3494, 37946, 41998, 42568, 45255, 4611, 5327,		
						6532, 7390, 9195		
*DDX3X*	Ovarian cancer	Poor	Post progression survival	<0.001	1435	GSE: 14764, 15622, 18520, 19829, 23554, 26193,	Array	201210_at
						26712, 27651, 30161, 3149, 51373, 63885, 65986,	RNA Seq	
						9891, TCGA (*n* = 565)		
*DDX3X*	Lung cancer	Good	Post progression survival	0.015	344	CAARRAY, GSE: 14814, 19188, 29013, 30219,	Array	201210_at
						31210, 3141, 31908, 37745, 43580, 4573, 50081,	RNA Seq	
						8894, TCGA (*n* = 133)		
*DDX3X*	Gastric cancer	Good	Post progression survival	<0.001	499	GSE: 14210, 15459, 22377, 29272, 51105, 62254	Array	201210_at

Survival data was collected from databases SurvExpress, TCGA, and Kaplan–Meier plotter. N.S.—no significance. “-”—no statistically significance.

**Table 2:** The correlation of *DDX3Y* with cancer patient survival.

Symbol	Cancer Type	Prognosis	Endpoint	*p* Value	Case	Dataset	Method	Probe ID
*DDX3Y*	Glioma	-	Overall survival	N.S.	153	TCGA	RNA Seq	
*DDX3Y*	Thyroid Cancer	Poor	Overall survival	0.027	501	TCGA	RNA Seq	
*DDX3Y*	Lung Cancer	Poor	Overall survival	0.047	994	TCGA	RNA Seq	
*DDX3Y*	Colorectal Cancer	Poor	Overall survival	0.039	597	TCGA	RNA Seq	
*DDX3Y*	Head and Neck Cancer	Good	Overall survival	<0.001	499	TCGA	RNA Seq	
*DDX3Y*	Stomach Cancer	Poor	Overall survival	0.003	354	TCGA	RNA Seq	
*DDX3Y*	Liver Cancer	-	Overall survival	N.S.	365	TCGA	RNA Seq	
*DDX3Y*	Pancreatic Cancer	Good	Overall survival	0.042	176	TCGA	RNA Seq	
*DDX3Y*	Renal Cancer	-	Overall survival	N.S.	877	TCGA	RNA Seq	
*DDX3Y*	Urothelial Cancer	-	Overall survival	N.S.	406	TCGA	RNA Seq	
*DDX3Y*	Prostate Cancer	Poor	Overall survival	0.034	494	TCGA	RNA Seq	
*DDX3Y*	Testis Cancer	-	Overall survival	N.S.	134	TCGA	RNA Seq	
*DDX3Y*	Breast cancer	N/A	Overall survival	N/A	1075	TCGA	RNA Seq	
*DDX3Y*	Cervical Cancer	N/A	Overall survival	N/A	291	TCGA	RNA Seq	
*DDX3Y*	Endometrial Cancer	N/A	Overall survival	N/A	541	TCGA	RNA Seq	
*DDX3Y*	Ovarian Cancer	N/A	Overall survival	N/A	373	TCGA	RNA Seq	
*DDX3Y*	Melanoma	Poor	Overall survival	0.022	102	TCGA	RNA Seq	
*DDX3Y*	Breast cancer	N/A	Relapse-free survival	N/A	3951	E-MTAB-365, E-TABM-43, GSE: 11121, 12093,	Array	205001_s_at
						12276, 1456, 16391, 16446, 16716, 17705, 17907,		
						18728, 19615, 20194, 20271, 2034, 20685, 20711,		
						21653, 2603, 26971, 2990, 31448, 31519, 32646,		
						3494, 37946, 41998, 42568, 45255, 4611, 5327,		
						6532, 7390, 9195		
*DDX3Y*	Ovarian cancer	N/A	Post progression survival	N/A	1435	GSE: 14764, 15622, 18520, 19829, 23554, 26193,	Array	205001_s_at
						26712, 27651, 30161, 3149, 51373, 63885, 65986,	RNA Seq	
						9891, TCGA (*n* = 565)		
*DDX3Y*	Lung cancer	-	Post progression survival	N.S.	344	CAARRAY, GSE: 14814, 19188, 29013, 30219,	Array	205001_s_at
						31210, 3141, 31908, 37745, 43580, 4573, 50081,	RNA Seq	
						8894, TCGA (*n* = 133)		
*DDX3Y*	Gastric cancer	Good	Post progression survival	<0.001	499	GSE: 14210, 15459, 22377, 29272, 51105, 62254	Array	205001_s_at

Survival data was collected from database SurvExpress, TCGA, and Kaplan–Meier plotter. N.S.—no significance. N/A: a collection of DDX3Y data is not available from female tissues. “-”—no statistically significance.
